# Complex Regional Pain Syndrome Triggered by Snake Bite: Diagnostic Utility of Triple-Phase Single-Photon Emission Computed Tomography-Computed Tomography Bone Scan—Case Report

**DOI:** 10.1055/s-0045-1812054

**Published:** 2025-10-04

**Authors:** Osama Ragab, Haneen Alqattan, Manar Al Daas, Hany M. A. Elrahman

**Affiliations:** 1 Department of Nuclear Medicine, Al-Adan Hospital, Ministry of Health, Hadiya, Kuwait

**Keywords:** complex regional pain syndrome, snake bite, MRI, bone scan, SPECT/CT

## Abstract

Complex regional pain syndrome (CRPS) is a chronic pain disorder with complex pathophysiology involving neurogenic inflammation and autonomic dysfunction. While CRPS is typically associated with trauma or surgery, its occurrence following a snakebite is extremely rare. We report the case of a 47-year-old man who developed persistent pain, swelling, and hypersensitivity in the left foot after a snakebite to the dorsum. Despite prompt administration antivenom and admission to the intensive care unit, the symptoms progressed without systemic infection. Magnetic resonance imaging (MRI) revealed evolving soft tissue inflammation, fat necrosis, and periostitis. Triple-phase bone scintigraphy with single-photon emission computed tomography/computed tomography (SPECT/CT) demonstrated increased perfusion and periarticular tracer uptake consistent with CRPS, while effectively excluding osteomyelitis and abscess. The diagnosis was established using the Budapest criteria, supported by clinical assessment and multimodal imaging, including evidence of evolving periostitis without cortical bone destruction. This case underscores the diagnostic challenge of CRPS in the absence of infection or trauma and highlights the crucial role of MRI and triple-phase bone scintigraphy with SPECT/CT in detecting early osseous involvement and guiding appropriate management.

## Introduction

Complex regional pain syndrome (CRPS) is a chronic pain condition characterized by Hyperalgesia and allodynia, typically affecting the extremities. 1 While the exact pathophysiology of CRPS remains under investigation, it is believed to involve dysfunction of both the central and peripheral nervous systems. 2 It remains unclear whether CRPS is primarily triggered by nerve damage or soft tissue injury. Recent research suggests that multiple mechanisms may contribute to its development. 3 CRPS following a snake bite is extremely rare, with only two cases reported globally. 4 5 de Mos et al. proposed that dysfunction in the autonomic and somatic nervous systems, along with neurogenic inflammation, tissue hypoxia, and psychological factors, may all play a role in CRPS pathogenesis. 6 The inflammation and edema resulting from a snake bite could potentially lead to the onset of CRPS. 7 Diagnosing CRPS is challenging and relies on evolving clinical criteria. Over the past two decades, these criteria have undergone several revisions. While laboratory tests and imaging techniques such as plain film radiography, magnetic resonance imaging (MRI), and triple-phase bone scan can aid in diagnosis, no definitive diagnostic standard exists, making CRPS a complex clinical entity to identify. 8 9 10 11 In particular, the use of three-phase bone scintigraphy with SPECT/CT adds significant diagnostic value. It enables early detection of regional blood flow changes and periosteal reaction, as well as provides anatomical localization, which helps differentiate CRPS from osteomyelitis or other inflammatory conditions. This hybrid imaging modality is especially valuable when clinical findings are ambiguous or overlap with other musculoskeletal disorders.

## Case Report

**Fig. 1:**
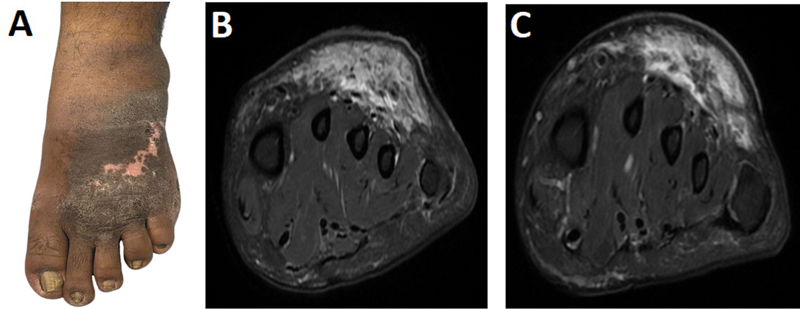
( **A** ) Clinical image showing skin changes and erythema. ( **B** ) Axial T2 MRI (January 2025) reveals soft tissue edema and fluid collection suggestive of cellulitis and abscess. ( **C** ) Follow-up MRI axial T2 (April 2025) shows persistent inflammation with new fat necrosis and deeper extension. MRI, magnetic resonance imaging.

A 47-year-old previously healthy male presented following a snake bite in October 2024, which occurred on the dorsum of his left foot. Initial symptoms included severe pain, swelling, erythema, and the formation of large bullae. He received prompt medical attention, including administration of antivenom and admission to the intensive care unit for close monitoring. Despite appropriate treatment, the patient continued to experience unresolved pain and swelling in the affected limb as shown in Fig. 1A . Over time, he reported worsening localized pain, warmth, and hypersensitivity, particularly during movement, no significant systemic symptoms were noted.

## Management and Imaging

**Fig. 2:**
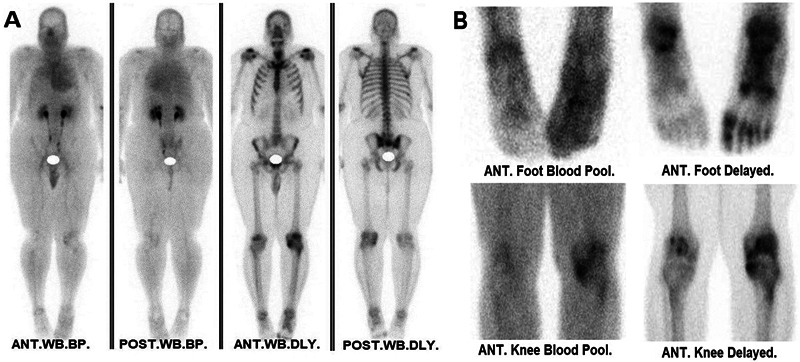
Bone scintigraphy. ( **A** ) Whole-body blood pool and delayed phase images demonstrating areas of increased tracer uptake. ( **B** ) Static blood pool and delayed images focusing on the anterior foot and left knee. Notably, the focal uptake in the medial tibial plateau of the left knee may suggest underlying arthritis rather than CRPS, which typically presents as a more diffuse periarticular uptake pattern. CRPS, complex regional pain syndrome.

**Fig. 3:**
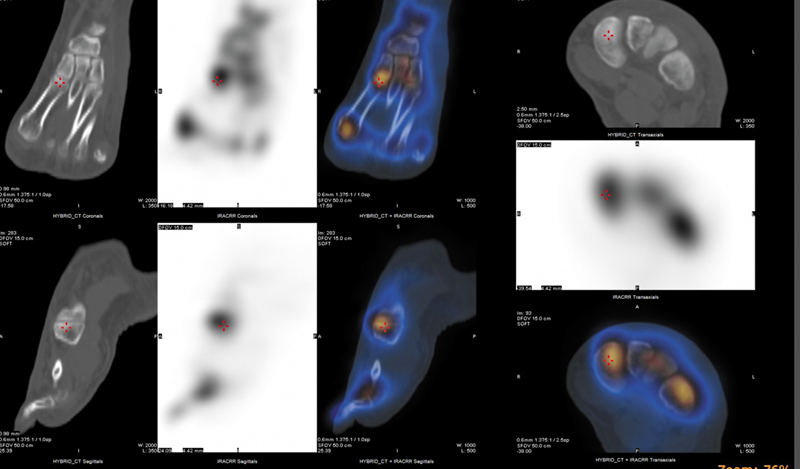
SPECT/CT imaging of the left foot. The figure includes CT images, nuclear medicine images, and fused hybrid SPECT/CT images, demonstrating localized tracer uptake and corresponding anatomical abnormalities in the affected region. CT, computed tomography; SPECT, single-photon emission computed tomography.

The patient's physical examination revealed signs of local inflammation and tenderness, raising concerns for cellulitis or abscess formation. An MRI was performed in January 2025, which showed diffuse subcutaneous soft tissue edema at the dorsum of the left foot with fluid collection as shown in Fig. 1B . There was a small soft tissue lesion (11 × 15 mm) at the first metatarsophalangeal joint space. The radiological impression suggested cellulitis with abscess formation and a potential diagnosis of fibromatosis. No evidence of fracture or joint derangement was identified. A follow-up MRI was conducted in April 2025 to assess for evolving pathology. The MRI revealed near-stable soft tissue signal changes, but with newly noted areas of fat necrosis, persistent irregular enhancement, and deeper extension of inflammation into the plantar aspect of the foot as shown in Fig. 1C . Signs of evolving periostitis throughout the metatarsal heads and necks were noted, along with synovial enhancement and joint effusion at the metatarsophalangeal joints. The radiologist recommended correlation with bone scan to evaluate for possible osteomyelitis. Following the last MRI in April 2025, a triple-phase bone scintigraphy was performed using ^99m^ Tc-HDP as per standard protocol. 12 Early-phase dynamic and blood pool scintigraphy revealed increased perfusion and hyperemia predominantly involving the knee and the dorsum of the left foot, indicating active inflammatory changes. The activity appeared diffusely distributed across multiple joint regions, suggesting an extensive inflammatory process. Delayed whole-body and SPECT/CT imaging of the feet showed diffuse peri-articular and articular tracer uptake in multiple joints of the left foot, specifically involving the left tibiotalar, tibiofibular, talonavicular, and various proximal and distal tarsal and metatarsal joints, with notable uptake at the first metatarsophalangeal joint, consistent with periostitis and elevated osteoblastic activity ( Fig. 2 ). Corresponding CT images revealed fat stranding and soft tissue edema localized to the left foot, further supporting an inflammatory etiology ( Fig. 3 ). Hybrid SPECT/CT imaging allowed precise anatomical localization of radiotracer uptake to bony structures and helped exclude abscess or osteolytic lesions, thereby enhancing diagnostic confidence. These findings collectively raise strong suspicion CRPS, affecting the left foot.

## Discussion

CRPS following a snakebite is an extremely rare clinical entity, with very few cases reported in the literature. In the case described by Pachowicz et al., 13 a patient developed CRPS in the same limb that had sustained a viper bite; however, bone scintigraphy was performed 2 years after the envenomation. Although the viper species was not identified and a direct causal relationship could not be definitively established, the authors proposed that the envenomation may have acted as a predisposing or facilitating factor by triggering a delayed inflammatory and autonomic response. In contrast, in our case, bone scintigraphy was performed 6 months after the snake bite, allowing for an earlier diagnosis of CRPS and strengthening the link between the envenomation and the onset of the syndrome.

CRPS typically progresses through three clinical phases. The first, known as the hyperemic phase, is marked by symptoms such as pain, tenderness, swelling (edema), increased skin temperature, excessive sweating, redness, and accelerated growth of hair and nails. The second phase, referred to as the dystrophic phase, usually emerges after several weeks and is characterized by persistent burning pain, hyperalgesia, cold and pale skin, hair loss, cyanosis, and trophic changes in the nails. In the third and final phase, the atrophic phase, which develops after several months, patients often experience muscle atrophy, joint contractures, and significant movement limitations. 2 Temperature asymmetry is a hallmark of CRPS, and thermography can serve as a useful diagnostic tool. A temperature difference of at least 1°C between affected and unaffected limbs is generally considered clinically significant. 14 In this case, it is consistent with the second phase of CRPS ( Fig. 1A ). The envenomation appears to have triggered a cascade of local inflammation and possible autonomic dysregulation, culminating in chronic pain, edema, and tissue changes. 2 The delayed yet progressive onset of symptoms, including swelling, hypersensitivity, and functional impairment in the absence of overt infection, supports this rare diagnosis. Imaging modalities played a critical role in confirming the diagnosis and excluding other potential causes. Triple-phase bone scan with SPECT/CT was particularly pivotal in detecting early alterations in bone metabolism that were not visible on conventional radiography. 10 This imaging modality confirmed active inflammatory involvement suggestive of CRPS and helped differentiate it from other conditions such as osteomyelitis or soft tissue infection. Furthermore, the bone scan supported MRI findings of evolving periostitis without cortical bone destruction, allowing for the confident exclusion of aggressive infectious processes.

The diagnosis of CRPS was made based on the Budapest criteria established by the International Association for the Study of Pain. 15 This was supported by the patient's disproportionate pain, sensory disturbances, vasomotor changes, edema, and the exclusion of alternative diagnoses through clinical assessment and multimodal imaging. This combination of functional and structural imaging provided valuable insight into the underlying neuroinflammatory changes, thereby guiding appropriate management. This case contributes to the limited body of literature documenting CRPS following snakebite envenomation and highlights the importance of considering this diagnosis in patients with persistent pain and swelling postenvenomation. Early recognition and the use of comprehensive imaging strategies, including triple-phase bone scan with SPECT/CT and MRI, are essential for accurate diagnosis and tailored therapeutic intervention.

## Conclusion

This case underscores the rare occurrence of CRPS following a snake bite, the patient had ongoing pain and swelling without signs of infection or fracture, making diagnosis difficult. Imaging with MRI and triple-phase bone scan with SPECT/CT helped detect early bone changes and rule out other conditions like infection. This highlights the importance of considering CRPS in similar cases and using advanced imaging to guide early and proper treatment.
